# Geohelminth Infections among Pregnant Women in Rural Western Kenya; a Cross-Sectional Study

**DOI:** 10.1371/journal.pntd.0000370

**Published:** 2009-01-27

**Authors:** Anna M. van Eijk, Kim A. Lindblade, Frank Odhiambo, Elizabeth Peterson, Daniel H. Rosen, Diana Karanja, John G. Ayisi, Ya Ping Shi, Kubaje Adazu, Laurence Slutsker

**Affiliations:** 1 Department of Infectious Diseases, Tropical Medicine and AIDS, Academic Medical Centre, University of Amsterdam, Amsterdam, The Netherlands; 2 Division of Parasitic Diseases, National Centre for Zoonotic, Vector-Borne and Enteric Diseases, Centers for Disease Control and Prevention, Atlanta, Georgia, United States of America; 3 Centre for Global Health Research, Kenya Medical Research Institute, Kisumu, Kenya; 4 Vermont Department of Health, Burlington, Vermont, United States of America; 5 Global AIDS Program, Centers for Disease Control and Prevention, Harare, Zimbabwe; George Washington University, United States of America

## Abstract

**Background:**

Geohelminth infections are common in rural western Kenya, but risk factors and effects among pregnant women are not clear.

**Methodology:**

During a community-based cross-sectional survey, pregnant women were interviewed and asked to provide a blood sample and a single fecal sample. Hemoglobin was measured and a blood slide examined for malaria. Geohelminth infections were identified using the concentration and Kato-Katz method.

**Results:**

Among 390 participants who provided a stool sample, 76.2% were infected with at least one geohelminth: 52.3% with *Ascaris lumbricoides*, 39.5% with hookworm, and 29.0% with *Trichuris trichiura*. Infection with at least one geohelminth species was associated with the use of an unprotected water source (adjusted odds ratio [AOR] 1.8, 95% confidence interval [CI] 1.1–3.0) and the lack of treatment of drinking water (AOR 1.8, 95% CI 1.1–3.1). Geohelminth infections were not associated with clinical symptoms, or low body mass index. A hookworm infection was associated with a lower mid upper arm circumference (adjusted mean decrease 0.7 cm, 95% CI 0.3–1.2 cm). Hookworm infections with an egg count ≥1000/gram feces (11 women) were associated with lower hemoglobin (adjusted mean decrease 1.5 g/dl, 95% CI 0.3–2.7). Among gravidae 2 and 3, women with *A. lumbricoides* were less likely to have malaria parasitemia (OR 0.4, 95% CI 0.2–0.8) compared to women without *A. lumbricoides*, unlike other gravidity groups.

**Conclusion:**

Geohelminth infections are common in this pregnant population; however, there were few observed detrimental effects. Routine provision of antihelminth treatment during an antenatal clinic visit is recommended, but in this area an evaluation of the impact on pregnancy, malaria, and birth outcome is useful.

## Author Summary

In rural western Kenya, both malaria and intestinal infections with worms are common. Pregnant women are particularly vulnerable to infection with malaria, but the effect on pregnancy of intestinal infections with worms is not clear and may depend both on how heavy the worm infection is and on the type of worm. Additionally, it is not clear whether infections with worms may affect malaria infections. In this article, we begin to disentangle some of these issues. Intestinal infections with worms were diagnosed in three-quarters of 390 pregnant women in western Kenya who provided a stool sample. In these women, intestinal worm infections caused a modest decrease both in haemoglobin levels and indicators of nutritional status. Women in their second and third pregnancies who were diagnosed with one particular type of worm infection (*Ascaris lumbricoides*) were less likely to have malaria than other women in their second or third pregnancies who did not have this type of worm infection. Although our results suggest that it would be good advice to treat women with drugs for intestinal worm infections during their pregnancy in this area, the effect on maternal and infant health and malaria infection needs further study.

## Introduction

Geohelminth infections are a major global health burden, with an estimated 3.8 billion persons infected, 720 million clinical cases, and an estimated 135,000 deaths attributed to clinical complications annually [1]. Geohelminth infections in pregnancy have been associated with iron deficiency, maternal anemia, and impaired nutritional status, as well as decreased infant birth weight, intra-uterine growth retardation, and adverse birth outcomes [1]–[6]. However, effects of geohelminths among pregnant women may differ by area and helminth burden [7],[8].

Hookworm disease is caused by *Ancylostoma duodenale* and *Necator americanus*. Mature hookworms can cause intestinal bleeding and protein loss proportional to worm burden; however, the severity of the effect is dependent on the host's underlying nutritional status [9]. Hookworm infections can cause or exacerbate iron deficiency and anemia. Blood loss can be a feature of *Trichuris trichiura* infection, but it is less prominent than in hookworm infection; however, it often occurs along with hookworm infections and so may accelerate the onset of iron-deficiency anemia. *Ascaris lumbricoides* infections are commonly asymptomatic, although clinical complications of extra-intestinal or high numbers of ascarids have been well described [10]. *A. lumbricoides* infection has been associated with impaired fat digestion, reduced vitamin absorption, and temporary lactose intolerance, and treatment has shown to improve nutritional status [1],[11]. Immunological effects of geohelminths can differ by species and may affect both a pregnant woman and her fetus [12]–[14].

The overlapping geographic distributions of geohelminth infections and malaria result in a high rate of co-infection [15]. There is conflicting information on interactions between *A. lumbricoides* and *Plasmodium falciparum*; studies have shown either an increase in clinical malaria in the presence of *A. lumbricoides* [16],[17], a protection from severe malaria [18],[19], or no observed interaction [20]. Pregnancy is known to alter the immune response of women to malaria; in holoendemic areas this results in an increase of mainly asymptomatic malaria during pregnancy. Recently, pregnancy has been associated with an increase in prevalence of *A. lumbricoides* and *T. trichiura* infections compared to non-pregnant women [21]. It is not clear if there is an interaction between geohelminth infections, particularly *A. lumbricoides*, and (asymptomatic) malaria in pregnancy [22].

A community-based cross-sectional survey of health and nutritional indicators among pregnant women in rural western Kenya allowed us to measure the prevalence of and to identify risk factors for geohelminth infections among pregnant women. We also examined effects of geohelminths on symptoms, anemia, and nutritional status, and explored the association of geohelminth infections with malaria.

## Methods

### Participants

The study was conducted in Gem (Wagai and Yala Divisions), Nyanza Province, western Kenya. Gem has a population of approximately 75,000 people residing in 142 villages. The vast majority of people are of Luo ethnicity (97.7%). This area is part of a health and demographic surveillance system. All births, deaths, pregnancies, in and out migrations, socio-economic information and educational level are regularly recorded and updated every four months. Based on previous estimates, we anticipated that at least 600 women in Gem would be pregnant at the time of the survey. All households received free insecticide-treated nets as part of a community-based trial (1998–2002) and continue to receive free retreatment [23]. There are two rainy seasons: the long rains take place from March to May and the short rains from October to December. Malaria is holoendemic and transmission occurs throughout the year, although insecticide-treated nets have reduced transmission by 90% [24].

### Description of Procedures

As part of the health and demographic surveillance system, one or two health workers in each village assist in the collection of information on births and deaths. For this survey, village health workers invited all pregnant women to a central location in a village on a specified day. Women were asked to bring their antenatal card if they had one, and a fresh (<24 hours old) stool sample in a container provided by the study. Only pregnant women who were living in the demographic surveillance area and were willing to give informed consent could participate.

A 5-day training workshop for all team members was held prior to the survey, which took place in July 2003, to standardize assessment methodology. Study staff administered a standardized questionnaire on medical and obstetric history, and blood was obtained for a malaria smear and a hemoglobin measurement. Weight was measured without shoes to the nearest 0.1 kg on a weighing scale and height was measured to the nearest centimeter on a locally made height instrument. The mid upper arm circumference (MUAC) was measured to the nearest 0.1 cm at the midpoint of the upper arm with an insertion tape, and axillary temperature was measured using a digital thermometer. Two independent observers performed all body measurements, and the average was used for data analysis. Socio-economic information at the household level was obtained from the health and demographic surveillance system. We used principal components analysis to generate weights for the following broad household characteristics: occupation of participant and spouse, source and quality of water, source of fuel for cooking, livestock and asset ownership, and dwelling/housing structure. From this we derived a wealth index, and scores were used to rank the study participants into socio-economic status quintiles [25].

All pregnant women received hematinic supplementation (200 mg ferrous sulfate 3 times per day and 5 mg folic acid once per day) for 30 days and, if appropriate, a dose of sulfadoxine-pyrimethamine as intermittent preventive treatment for malaria as per national guidelines. A clinical officer and a nurse treated additional complaints. All participants in their second or third trimester with a geohelminth infection received a single treatment dose of 400 mg albendazole.

### Laboratory

Thick and thin smears were stained with Giemsa and examined under oil immersion; parasite counts were determined against 500 white blood cells, assuming a white blood cell count of 8000/µl, and species were identified. A 10% sample of smears was reread for quality control. Hemoglobin was measured using a Hemocue hemoglobin detection system (HemoCue AB, Angelholm, Sweden) on site. Stool samples were collected and microscopically examined for geohelminth infection using a modification of the formol-ether and ethyl acetate by concentration technique and by Kato-Katz method; hookworm egg counts were performed [26],[27]. The stool results used were a combination of both methods; if a geohelminth was identified by either method or both methods, the geohelminth was considered present in that stool sample.

### Ethics

The study protocol was reviewed and approved by the Kenya Medical Research Institute (Nairobi, Kenya) and the Centers for Disease Control and Prevention (Atlanta, USA).

All participants in this study gave written informed consent or indicated their approval with a thumb print in the presence of a witness if the participant was illiterate.

### Definitions

Malaria was defined as the presence of any asexual blood stage parasite of any species in a thick smear independent of signs or symptoms. The body mass index was calculated as weight in kilograms/height (in meters) squared; a low body mass or underweight was defined as a body mass index less than the average 5^th^ percentile of the body mass index by trimester of a Swiss reference population (18.8 kg/m^2^ for the first trimester, 20.2 kg/m^2^ for the second and 22.3 kg/m^2^ for the third trimester) [28]. A low MUAC or wasting was defined as a mid upper arm circumference less than 22 cm [29]. A protected water source for drinking water included a borehole or a protected spring; an unprotected water source included surface water (stream, river, lake or rainwater) or an unprotected spring. Treatment of drinking water in the household was defined as boiling, filtering or treatment with alum or chlorine. A high socioeconomic status was defined as the upper two quintiles of the wealth index.

### Statistical methods

Differences in proportions were analyzed using the chi-square test or Fisher's exact test when appropriate. Differences in means were compared by Student's *t*-test or a non-parametric test if not normally distributed. The following variables were examined for their association with geohelminth infection (overall and by species as dependent variable): socio-economic status, level of education, type of water source, household water treatment, marital status, young age, gravidity, trimester of pregnancy, malaria, a report of soil eating, and presence of animals in the house. In addition, in the models for separate geohelminth species, the association with the presence of other geohelminth species was examined as well. Odds ratios and their 95% confidence intervals (CI) were calculated. Adjusted odds ratios were obtained using a logistic regression whereby all factors significant in the univariate analyses were introduced (as independent variables), and factors with a P-value>0.05 were removed. However, we kept a malaria infection in the model for each geohelminth species, even if not significant, because this was our variable of interest. Linear regression was used to assess the relationship between geohelminths and both hemoglobin and MUAC. We examined effects and prevalence of geohelminths by gravidity group as well, whereby we divided gravidity group into primigravidae, gravidae 2 and 3, and gravidae ≥4. We decided for this division after inspection of the graphs of prevalence of geohelminths by gravidity, and because of our interest in the interaction of geohelminth infections with malaria. Malaria is known to be more common among primigravidae, and to decrease with increasing gravidity number in this area [30]. SAS (SAS system for Windows version 8, SAS, Cary, NC) was used for all analyses. A two-sided P-value<0.05 was considered statistically significant.

## Results

### Characteristics of the study population

Of the 673 pregnant women who participated, 390 women brought stool samples (58.0%). Participants who brought a stool sample were older (median age 25, interquartile range [IQR] 21–31 years) than women who did not bring a stool sample (median age 24, IQR 21–29 years, Mann-Whitney U test *P* = 0.009), and were more likely to be of higher gravidity number, and of lower socio-economic status and education level (Table 1). Women who brought stool samples were more likely to use protected water sources, but were also more likely to report diarrhea in the 2 weeks previous to the survey (Table 1). There was no difference in other reported symptoms, such as abdominal pain (90.3% in total population), loss of appetite (69.8%), fever (62.6%), cough (57.2%), itching (33.8%), or rash (20.1%). During the current pregnancy, none of the participants reported the use of antihelminth medication.

**Table 1:** Characteristics of the participating pregnant women by presence of a stool sample result, Gem, July 2003.

	Stool present, % (N = 390)	Stool absent, % (N = 283)	Total % (N = 673)
Age			
<20 years	15.9*	15.6	15.8
20–24 years	28.5	36.8	31.9
25–29 years	22.1	24.0	22.9
≥30 years	33.6	23.7	29.4
Gravidity			
Gravidae 1	11.5*	15.2	13.1
Gravidae 2 and 3	32.1	37.8	34.5
Gravidae ≥4	56.4	47.0	52.5
1^st^ or 2^nd^ trimester of pregnancy †	47.8	52.7	49.9
Married	86.7	86.2	86.5
Low/medium SES †	71.5*	63.0	68.1
<8 yrs of education †	63.5*	52.6	59.0
Use of unprotected water source †	59.3*	73.3	65.0
Drinking water untreated †	63.3	64.7	63.9
History of soil eating	46.9	40.6	44.3
History of diarrhea in last 2 weeks	21.3*	14.5	18.4
MUAC <22 cm	3.1	1.8	2.5
Low body mass index †	19.4	19.8	19.6
Malaria	37.8	33.2	35.9
Hemoglobin <11 g/dl	51.8	54.1	52.8
Antenatal clinic visited	38.5	44.9	41.2

Abbreviations: SES: socio-economic status; MUAC: mid-upper arm circumference.

*: Chi-square test *P*<0.05 comparing characteristic of women with vs. without a stool sample.

**†:** Trimester of pregnancy missing for 3 women; SES missing for 156 women (23.2%); years of education missing for 110 women (16.3%); water source and treatment missing for 133 women (19.8%); body mass index missing for 3 women.

### Geohelminth infections: prevalence and effects

**Figure 1:**
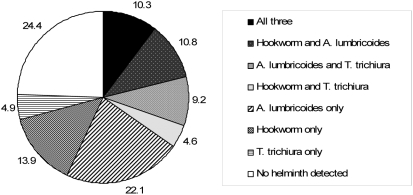
Prevalence (%) of geohelminth infections among 390 pregnant women, Gem, July 2003.

**Figure 2:**
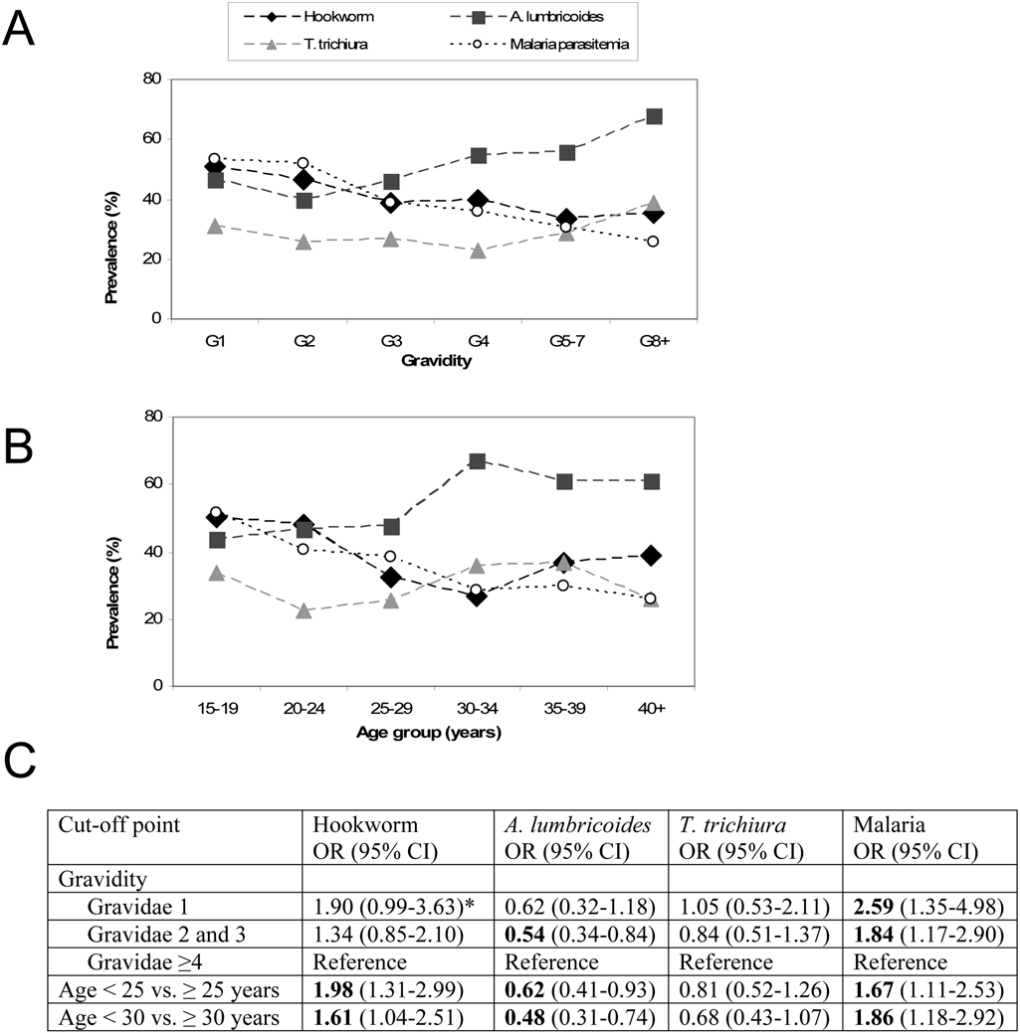
Prevalence of geohelminth infections and odds ratios by gravidity and age group among 390 pregnant women, Gem, July 2003. OR: odds ratio; CI: confidence interval. Significant odds ratios are printed in bold. Malaria parasitemia is presented for comparison. The cut-off points for age and gravidity were obtained from visual inspection of the graphs above. * *P* = 0.05.

Overall, 76.2% of the 390 women who provided a stool sample were infected with a geohelminth (Figure 1). *A. lumbricoides* (52.3%) was most prevalent, followed by hookworm (39.5%) and *T. trichiura* (29.0%). *Schistosoma mansoni* was detected in the stool of 2 women; *S. strongyloides* was not detected. Overall, hookworm infections were low density, with egg counts of ≥1000 eggs/gram feces in only 7.1% of infections. Patterns of the prevalence of geohelminth infections seemed more distinct by gravidity compared to age (Figure 2A and 2B). Increased gravidity number was associated with increased prevalence of *A. lumbricoides* and decreased prevalence of hookworm infections (trend test *P* = 0.003 and *P* = 0.03, respectively); there was no clear pattern for *T. trichiura* (Figure 2C).

Complaints in the previous two weeks such as diarrhea, loss of appetite, abdominal pain, itching, fever, cough, or a rash were not associated with geohelminth infections overall, or by species (data not shown), or by combination of species. Geohelminth infections overall, or by species or combination of species, were not associated with a low body mass index or a hemoglobin <8 g/dl. *T. trichiura* was not associated with either hemoglobin or MUAC (Table 2). In linear regression, a hookworm infection was not associated with hemoglobin; however, infections of ≥1000 eggs/gram feces (11 women) were associated with a mean decrease of 1.5 g/dl in hemoglobin (95% CI 0.3–2.7 g/dl, compared to hemoglobin among women with no hookworm infection, adjusted for malaria, marital status, report of soil eating and treatment of drinking water).

**Table 2:** Effects of geohelminth infections on hemoglobin, anemia, and mid upper arm circumference by type of infection and gravida group among pregnant women in Gem, July 2003.

	Type of infection		
	Hookworm	*A. lumbricoides*	*T. trichiura*
	Hemoglobin*, mean difference (95% CI) g/dl		
All women	−0.18 (−0.60 to 0.25)	−0.23 (−0.65 to 0.18)	0.12 (−0.34 to 0.58)
Gravidae 1	0.76 (−0.29 to 1.80)	**−1.37** (−2.3 to −0.43)	−0.32 (−1.43 to 0.79)
Gravidae 2 and 3	−0.38 (−1.14 to 0.37)	0.47 (−0.32 to 1.25)	−0.17 (−1.03 to 0.68)
Gravidae ≥4	−0.24 (−0.83 to 0.34)	−0.33 (−0.90 to 0.24)	0.25 (−0.39 to 0.88)
	Anemia: Hemoglobin <11 g/dl* AOR (95% CI)		
All women	1.03 (0.63–1.68)	1.14 (0.71–1.84)	1.14 (0.67–1.94)
Gravidae 1	0.98 (0.17–5.63) †	**7.32** (1.08–49.63) †	4.54 (0.42–49.07) †
Gravidae 2 and 3	1.20 (0.48–3.01)	0.86 (0.33–2.22)	1.66 (0.59–4.64)
Gravidae ≥4	0.99 (0.51–1.91)	1.01 (0.53–1.91)	0.92 (0.45–1.88)
	MUAC‡, mean difference (95% CI), cm		
All women	**−0.75** (−1.23 to −0.27)	−0.06 (−0.41 to 0.53)	0.09 (−0.44 to 0.61)
Gravidae 1	**−1.41** (−2.66 to −0.16)	0.02 (−1.15 to 1.19)	−1.02 (−2.36 to 0.33)
Gravidae 2 and 3	−0.58 (−1.33 to 0.17)	0.07 (−0.67 to 0.82)	−0.21 (−1.05 to 0.63)
Gravidae ≥4	−0.50 (−1.18 to 0.19)	−0.20 (−0.89 to 0.49)	0.53 (−0.21 to 1.28)

Abbreviations: AOR: adjusted odds ratio; CI: confidence interval; MUAC: mid upper arm circumference. Significant differences or odds ratios are printed in bold.

*: Models adjusted for malaria, marital status, treatment of water and a report of soil eating and other geohelminths unless indicated otherwise. Malaria, marital status, treatment of water and a report of soil eating were significantly associated with anemia and hemoglobin level in the model for all women, and for uniformity kept in the models by gravidity, even when not significant.

**†:** Model adjusted for malaria, water treatment and other geohelminths.

**‡:** Model only adjusted for other geohelminths.

By gravidity, hookworm infection was associated with a significant decrease in MUAC, mainly among primigravidae (Table 2). *A. lumbricoides* was associated with anemia and lower hemoglobin level among primigravidae (independent of hookworm), but not overall or in any other gravidity group (Table 2).

### Factors associated with geohelminth infections

Infections with at least one geohelminth species were associated with the use of an unprotected water source (adjusted odds ratio [AOR] 1.8, 95% CI 1.1–3.0) and the lack of household treatment of drinking water (AOR 1.8, 95% CI 1.1–3.1, adjusted for each other). However, water source or treatment was not statistically significantly associated with any geohelminth species. A report of eating soil was not associated with geohelminth infection overall or by species.

Factors associated with a hookworm infection included *T. trichiura* (increased risk) and being married (decreased risk) (Table 3). *T. trichiura* prevalence increased with higher hookworm load and was 54.6% among women with ≥1000 eggs/gram feces, 36.4% among women with 1–999 eggs/gram feces, and 23.3% among women with no hookworm infection (trend test *P* = 0.001).

**Table 3:** Factors associated with type of geohelminth infection in multivariate analysis among pregnant women, Gem, July 2003.

	Type of infection		
	Hookworm	*A. lumbricoides* *	*T. trichiura*
	AOR (95% CI)	AOR (95% CI)	AOR (95% CI)
1^st^ or 2^nd^ vs. 3^rd^ trimester	NS	1.62 (1.06–2.47)	NS
Age <30 years	1.79 (1.13–2.84)	0.52 (0.33–0.82)	NS
Married	0.42 (0.23–0.77)	NS	NS
*A. lumbricoides*	NS	NA	2.46 (1.54–3.93)
Hookworm	NA	NS	2.02 (1.28–3.19)
*T. trichuria*	2.11 (1.33–3.34)	2.41 (1.50–3.87)	NA
Malaria	1.00 (0.64–1.55)	0.68 (0.44–1.05)	1.37 (0.86–2.18)

Abbreviations: AOR: adjusted odd ratios; CI: confidence interval; NS: not significant; NA: not applicable. Variables are adjusted for the reported odds ratios in the same column.

*: Multivariate model with gravidity instead of age: Gravidae 1: AOR 0.70, 95% CI 0.36–1.37, gravidae 2 and 3: AOR 0.56, 95% CI 0.35–0.89, gravidae ≥4 as reference, malaria: AOR 0.68, 95% CI 0.44–1.05. Interaction term malaria and gravidae 2 and 3: *P* = 0.03.

Factors associated with an increased risk of infection with *A. lumbricoides* included being in the first or second trimester of pregnancy and a stool infection with *T. trichiura*; conversely, age <30 years (or in a separate model including gravidity instead of age being gravidae 2 or 3) were associated with a decreased risk (Table 3). For an infection with *T. trichiura*, both *A. lumbricoides* and hookworm infections were risk factors, but no other factor was significant.

### Geohelminth infections and malaria

**Figure 3:**
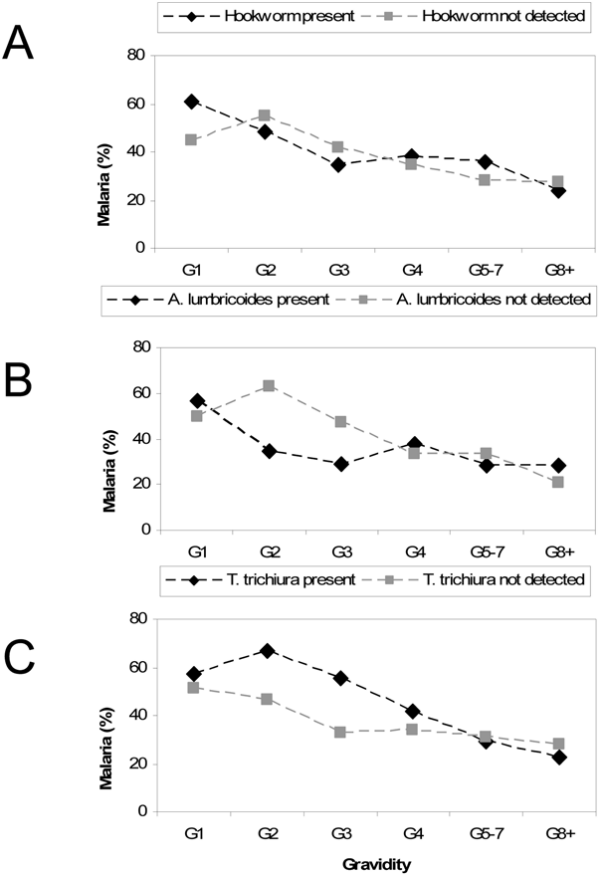
Prevalence of malaria by geohelminth infection and gravidity group among 390 pregnant women in Gem, July 2003.

Malaria was detected in the blood smear of 37.8% of the women: 95.9% of infections were *P. falciparum* only, with the remainder mixed infections. Three women had documented fever at the time of visit; none of them had malaria. A history of fever in the last two weeks (64.4% of women) was not associated with a positive blood smear. Prevalence of malaria by gravidity or age (Figure 2A and 2B) showed the familiar pattern of decreasing malaria prevalence with increasing gravidity or age. Patterns of malaria by gravidity in the presence or absence of geohelminth infections can be seen in Figure 3A–3C: Among gravidae 2 and 3, those with *A. lumbricoides* were less likely to have malaria (OR 0.4, 95% CI 0.2–0.8) than were those without *A. lumbricoides*; conversely, among gravidae 2 and 3, those with *T. trichiura* were more likely to have malaria (OR 2.39, 95% CI 1.06–5.40) than those without *T. trichiura*. These relationships were not observed in other gravidity groups.

In multivariate analysis, malaria was not associated with either hookworm or *T. trichiura* infection; for *A. lumbricoides*, there was a trend towards decreased malaria risk (AOR 0.7, 95% CI 0.4–1.0, *P* = 0.06, model adjusted for gravidity, marital status, and trimester of pregnancy). When combining gravidity and *A. lumbricoides* infection in one variable and introducing this in the model for factors associated with malaria infection, compared to gravidae ≥4 without *A. lumbricoides* infection, gravidae 2 and 3 without *A. lumbricoides* infection had an AOR of 2.5 (95% CI 1.3–4.7) for malaria, whereas the same group with *A. lumbricoides* had an AOR of 0.9 (95% CI 0.4–1.9) for malaria.

No interaction was noted between malaria and any geohelminth on hemoglobin.

## Discussion

Intestinal infections with *A. lumbricoides*, *T. trichiura* or hookworm were very common among pregnant women in rural Kenya; 3 out of 4 women had one or more parasites, and 1 out of 10 were infected with all 3. Of note, even these high prevalence figures may underestimate the true burden, because participants who did not bring a stool sample were more likely to use an unprotected water source, which was associated with a geohelminth infection in this survey. High geohelminth infection rates among pregnant women have been reported elsewhere; in Nepal (any geohelminth 89%, hookworm 74%) [2], in Peru (any geohelminth 90.7%, hookworm 63.9%) [31], in Uganda (any geohelminth 71%, hookworm 66.6%) [7], and coastal Kenya (hookworm 75%) [32].

The prevalence of hookworm infection has been described to increase throughout childhood followed by a stable plateau in adulthood [1],[20],[33]. Among pregnant women we noted a decrease in hookworm prevalence after approximately 25 years of age. The prevalence of *A. lumbricoides* and *T. trichiura* have been reported to peak in childhood and decline thereafter [1],[20],[33],[34]. Interestingly, we observed an increase of *A. lumbricoides* infections after around 30 years. It is possible that pregnancy status may have affected this outcome. Pregnancy has been associated with an increase in *A. lumbricoides* and *T. trichiura* infections compared to non-pregnant women in a small study in Gabon [21]. In a study among 1246 school children aged 10–12 years in a neighboring area conducted two years before the current study, there was a lower prevalence of *A. lumbricoides* and *T. trichiura* (mean *A. lumbricoides* prevalence over 32 schools: 22.3%, range 0–83.8%, and *T. trichiura*: 17.9%, range 5.1–64.9%); however, the prevalence of these two geohelminths was higher in the geographic location bordering our study area [35].

Geohelminth infections are transmitted through soil; however, in this study we did not find an association between soil eating and geohelminth infection, similar to findings from a study among pregnant women in Zanzibar [36]. We did see an association with drinking water source and infection. It is possible that the use of an unprotected water source and the absence of water treatment were markers for soil exposure; alternatively, transmission through water contaminated with soil may be an additional infection route. It is of note that none of the individual species was significantly associated with type of drinking water source or water treatment.

Although the prevalence of geohelminth infection was high, its effect of on maternal health was not straightforward. Compared to other studies, effects of hookworm infection on anemia and hemoglobin were limited, possibly because of the low density of infections [2],[7]. Other studies have reported a similar lack of effect on anemia in areas with a higher hookworm prevalence [8],[31]. Hookworm infection has been associated with inhibition of growth among children [11], and was associated with a lower MUAC in our study. In a study in Zanzibar, treatment of geohelminth infections with mebendazole among young children was associated with an improvement of anthropometric indicators; the authors speculated that geohelminth infection may stimulate inflammatory responses with deleterious effects on protein metabolism and erythropoiesis during first infections [37]. It is possible that pregnancy-related immunomodulation may enhance similar mechanisms. If so, the greatest effect would be expected in primigravidae; and indeed this is in accord with our observations for hookworm and *A. lumbricoides*.

The interactions between malaria and geohelminth infections have been a subject of recent interest with regards to assessment of vaccine efficacy [22], [38]–[41]. During pregnancy, malaria parasites sequester in the placenta by binding to novel receptors; women become more resistant to *P. falciparum* malaria with successive pregnancies as they acquire antibodies to these special placental forms of the parasite [42]. In areas of high malaria transmission, the increase in malaria during pregnancy is mainly asymptomatic [43]. Geohelminths may trigger a T helper-2 response leading to the production of non-cytophylic clinically non-effective antibodies [39], which may delay the development of an effective immune response to malaria in pregnancy. According to this hypothesis, we could expect an increase in malaria among women with geohelminth infections in their first pregnancies. Although our sample size was too small to explore fully the relationship between malaria and geohelminths by gravidity, we did note an increase in malaria among gravidae 2 and 3 infected with *T. trichiura* in univariate analysis, but not in multivariate analysis or among the other geohelminths. Of note, the effect of *A. lumbricoides* on asymptomatic malaria parasitemia among gravidae 2 and 3 was the opposite, and remained significant in multivariate analysis. This would suggest that an *A. lumbricoides* infection may either prevent or control the development or improve the clearance of a *P. falciparum* infection. An increase in *P. falciparum* densities was reported among children aged 5–14 years treated with levimasole when participating in a randomized controlled trial to evaluate antihelminth treatment [44], indicating that *A. lumbricoides* may affect the development of concomitant *P. falciparum* infections. Maeno et al. (1993) reported decreased sequestration of parasite infected red blood cells in the placentas of women with IgE deposits in the capillaries, which may be another indication of the negative interaction between a geohelminth and a malaria infection [45]. If we would assume a causal relationship between an *A. lumbricoides* and a malaria infection, the population attributable fraction of malaria among gravidae 2 and 3 in this area due to not having an *A. lumbricoides* infection could be approximately 30% and among the total pregnant population about 10%. Again, the sample size in our study is small, and our findings would need to be confirmed in larger studies. Given the differing effects, it may not be advisable to categorize geohelminths as a group when analyzing their interaction with malaria; among pregnant women it may be advisable to explore the interaction by gravidity group. Similar to an observation from coastal Kenya, we did not note an interaction between malaria and any geohelminth on hemoglobin among pregnant women [40].

### Limitations

Limitations of our study include the small sample size and use of a single stool specimen to assess infection status, which may have underestimated geohelminth burden. Also, we did not assess the HIV-status of the participants, and HIV-infected women are more likely to carry malaria parasites and to be anemic than HIV-uninfected women. In an adjacent rural area, an HIV prevalence of 21% has been described among women in the age group 13–34 years [30]. HIV infection was not associated with geohelminth infection in Ethiopia [46], but a lower prevalence of *A. lumbricoides* [47] or geohelminth infections overall among HIV-infected persons compared to HIV-uninfected persons has been described [48], raising the possibility that HIV infection may be a potential confounder of the association between geohelminths and malaria or anemia.

Mebendazole and albendazole are considered safe in pregnancy after the first trimester, and in areas with a high prevalence of hookworm infection (20–30% or more), anthelminth treatment once in the second trimester of pregnancy is recommended [1],[4],[49],[50]. Because 90% of pregnant women in this area attend ANC at least once [51], the delivery of routine antihelminth treatment in pregnancy through the antenatal clinic seems feasible. However, given the limited clinical impact of geohelminth infection during pregnancy observed here, as well as the potential complex interaction with malaria, more detailed studies in pregnant women, including birth outcome information, may be useful to assess the impact of routine prenatal use of geohelminth treatment in this area.
